# Fracture of the Carpal Extremity of the Ulna

**Published:** 1860-06

**Authors:** James H. Farris

**Affiliations:** Danville, Ill.


THE CHICAGO
### MEDICAL JOURNAL.

VOL. III. June, 1860. NO. 6.

### Original Communications.

### ARTICLE 1.

### FRACTURE OF THE CARPAL EXTREMITY OF THE ULNA,

### With Displacement of the Pisiform Bone, and Extirpation of the Latter Six Months After the Injury.

BY JAS. H. FARIS, M. D., DANVILLE, ILL.

May 2d, 1859, I was consulted by Mrs. Susan Palmer, relative to the above described injury, received the day before. By manipulation, I discovered an oblique fracture of the ulna immediately above its capitulum. The swelling was not excessive, crepitus evident, deformity quite apparent; patient complains of but little pain. She informed me that after raising a window she turned to leave it, and, while in the act of doing so, observed the sash falling; attempting to arrest it, she received the injury by the horizontal mullion coming in violent contact with the ulnar half of the wrist joint. The fragment was easily returned to its proper place by grasping it between the thumb and index finger of the left hand, and rotating hand from left to right. A splint extending from the elbow to the points of the fingers, placed on the back of the fore arm; one somewhat shorter applied in front, with the addition of a pad in the palm of the hand; a third along the inner side of the wrist joint; the application of a roller encircling the arm, with orders to keep the joint wet with cold water, constituted the dressing. In a few hours the patient complained of rapid darting pain in the course of the ulnar and median nerve, in the axilla, and side of the neck, as high as the origin of the brachial plexus.

The appliances were now removed without any relief whatever. The part was thoroughly examined, the fragments separated and again replaced; when the hand was flexed forward, she was free from pain; when backwards, the pain was excessive. I could not discover that a portion of the nerve had been caught by the fragments; there was no unusual depression or prominence by which I could be guided in my diagnosis, no external wound, not even an abrasion of the skin, indicated that a foreign body from without was producing the mischief. Was there laceration of the nerve? This could not occur, its anitomical relations to the superficial and deep seated tendons of the flexors forbiding such a conclusion.

I continued my attentions to this lady up to the 25th inst., during which period she suffered the most excruciating pain, with scarcely a moment of rest. Morphine in larger doses, liniments, volatile and sedative, variously combined, turpentine with ammonia, the latter with chloroform, tincture of aconite and belladonna, frigorific mixtures of salt and ice, spirits of wine added to diacetate of lead, and acetic acid, and finally a blister from the wrist to the middle of the neck; *none of which alleviated or aggravated the pain.*

June 15th, the constitution was sympathising to an alarming extent, and loss of rest and appetite, morosity of temper, the sides of the little finger and the ulnar side of the ring finger were almost useless; shrivelled, cold, and almost insensible to the prick of a pin; atrophy of the whole arm making its appearance; the circulation in the radial artery not affected, the ulnar could not be found.

Same treatment continued without amendment, until the 20th of June; Drs. Tithian, Scott, and Lemon met me in council; a number of prescriptions suggested, as many theories advanced, but no good resulting.

21st. During my manipulations I was satisfied that the pisiform bone was displaced and now resting upon the neck* of the front surface of the ulnar, thereby producing the irritation. A pit at the normal location of the bone, satisfied my mind, fully, that my diagnosis was correct, and my patient's suffering at about an end. In the evening the physicians were re-called, my convictions stated, and an explanatory incision proposed on the spot; and in the event the bone was not displaced, to remove an inch and a half of the ulnar nerve. Nothing was done, and I left the patient to be tortured by a repetition of extreme pain.

July 1st, I proposed taking her to Chicago, which I did. A course was prescribed, and upon my return home (July 7th) I pushed it with unremitting energy, until August 12th; aiding it with such adjuvants as I deemed necessary. At this time it was laid aside and a letter of inquiry directed to Prof. Daniel Brainard, of Chicago, who, in his reply, advised me to be influenced by sound judgment, and remove it "if I was sure it was misplaced." Again the physicians were convened, doubts of the propriety of an operation fully discussed, and fears of a suit for malpractice canvassed.

No course worthy of record was adopted from the 15th of August, until the 24th of October. During this long interval the patient was reduced to an alarming extent; each day telling the story of increasing constitutional debility, prominent nervous irritabiilty, and their usual attendants.

At 4 o'clock, P. M., October 24th., I performed the operation, (assisted by the physicians already mentioned) in the following manner:

The patient was seated in an arm chair with a high back, the right arm and hand were lashed fast to the corresponding arm of the chair, and a handkerchief saturated with chloro-

---

*By the neck, (for convenience of description) I mean that portion of the carpal extremity of the ulna immediately above its capitulum.—WILSON.

form held within an inch of the nostrils; unfortunately, at each inspiration, spasm of the glottis prevented its inhalation and it was laid aside. I now gave her ¼ of a grain of sulph. morph.; and half an hour after, stationed one Dr. in front, another, with a heavy towel passed around the chest including the left arm, a third secured the head, and a fourth placing himself on left side of the patient, he grasped with his left hand the right of the patient and arm of chair to which it was bound, and with the right laid hold of the arm below the elbow.

Seating myself on the right side of patient, I inserted my knife at a point two inches above the wrist joint, and a fourth of an inch to the ulnar side of the tendon of flexor carpi ulnaris, and carrying it towards the radius and down to the wrist joint in a semilunar direction; I terminated it on the ulnar side of the same tendon, above the original position of the pisiform. The flaps formed were turned inwards, the fascia and subcutaneous tissue removed and the bone found full three-fourths of an inch out of its natural position, resting within and above the styloid process and firmly adhering to the surrounding structures. Inserting a small blade flatwise, beneath the tendon from the radial side, I cut all the adhesions in that direction; reversing the knife, I passed it (flatwise) from within outwards and downwards, destroying the connecting tissue between the inner fasciculi of the flexor sublimis and bone. The tendons were separated by blunt hooks, and by a careful dissection the relation of the artery and nerve to the bone were made apparent; the artery passing along the radial side of the bone, presented a well marked crescent, evidently having been turned out of its course by the pressure of the bone; the articular facet of the bone was downwards, and the nerve passing between its centre and the portion of ulnar above described. Taking hold of it with a pair of strong forceps, I hoped to be able to evulse it, but being carious, it was crushed, and I was obliged to remove it in several pieces, which was done without losing half an ounce of blood, or doing injury to the vessel or nerve.

The hooks being removed, the tendons restored themselves to their natural position; the flaps were adjusted, a few strips of adhesive plaster applied, and the whole covered with roller and compress.

The operation occupied a period of twenty-two minutes. The patient was removed to a couch, where she sank into a quiet sleep, continuing nine hours.

October 25th—Patient cheerful, suffers some little from stiffness of shoulder joint, and a sense of tingling at the site of incision.

From this time onward she continued to improve locally and constitutionally. The incision healed by the first intention, and by the seventh day was completely cicatrized and firm. The pain along the nervous track became less and less daily, and has at this time, (April 27th, 1860,) entirely ceased.

Motion at the shoulder joint is not as perfect as it was prior to the injury; doubtless depending upon the position in which she carried her arm for six months. Motion at the elbow is good; at the wrist flexion and extension, normal.—The ulnar half of the hand is somewhat atrophied, the little and ring finger a little stiff, physical appearance otherwise healthy. To use the patient's own language, she "cannot bear to have pins stuck in her hand or fingers, and can carry as much wood and water with it as with the right."

The treatment after the operation, consisted in the application of cold water, (continuously) a few doses of mophine, and an occasional dose of pill cath. compo., to correct the secretions until the wound closed; after which, primarily, stimulating liniments; secondarily, dry, with flannel first, and after with the hand, then the flesh brush. The remainder of the time she has been using an electro-magnetic battery, with, I think, the happy efiect of arousing the muscles to action through the medium of the nervous system.

It is worthy of remark, that on the fifth day after the removal of this bone, the recuperative energies of the system were busily engaged. Constitutionally, the pulse became less frequent, the secretions active, tongue clean, and freedom from nervous restlessness and jactitation. Mentally she emerged from her taciturn irritability, to a pleasant and agreeable loquacity; receiving hei friends kindly, and making various explanations in regard to her comparative good health.

